# The CDK inhibitor Roscovitine enhances the therapeutic efficacy of anti-PD-1 in non-small cell lung cancer

**DOI:** 10.3389/fonc.2025.1745967

**Published:** 2026-01-05

**Authors:** C. Marcela Diaz-Montero, Elise G. Holvey-Bates, Patricia A. Rayman, Yvonne Parker, Daniel J. Lindner, George R. Stark, Sarmishtha De

**Affiliations:** 1 Center for Immunotherapy and Precision Immuno-Oncology (CITI), Cleveland Clinic, Cleveland, OH, United States; 2 Department of Cancer Biology, Cleveland Clinic Lerner Research Institute, Cleveland, OH, United States; 3 Translational Hematology and Oncology Research, Taussig Cancer Center, Cleveland Clinic, Cleveland, OH, United States

**Keywords:** circulating immune cells, combination therapy, immune modulation, non-small cell lung cancer, PD-L1, tumor-infiltrating immune cells

## Abstract

Immune checkpoint blockade (ICB) targeting the PD-1/PD-L1 axis has significantly improved outcomes in non-small cell lung cancer (NSCLC), yet many patients fail to respond. High PD-L1 expression, often predictive of response, paradoxically correlates with poor prognosis and immune suppression driven by the tumor microenvironment (TME), including myeloid-derived suppressor cells (MDSCs). Roscovitine (Seliciclib), a cyclin-dependent kinase (CDK) inhibitor, downregulates PD-L1 and exhibits immunomodulatory effects, but its potential to enhance ICB efficacy in NSCLC is unknown. Using a syngeneic, immune-competent Lewis lung carcinoma (LLC) mouse model, we evaluated the therapeutic impact of Roscovitine alone or combined with anti-PD-1 therapy. The combination substantially reduced tumor burden, prolonged survival, and induced durable anti-tumor immunity upon tumor re-challenge. Mechanistically, Roscovitine decreased PD-L1 expression on tumor cells and myeloid populations, including circulating and tumor-infiltrating MDSCs, while reducing CCR2^+^ MDSC frequency in circulation. This was accompanied by increased infiltration of cytotoxic CD8^+^ T cells and NK cells into the tumor, collectively enhancing anti-tumor immune activity within the TME. These findings demonstrate that Roscovitine potentiates anti-PD-1 therapy by simultaneously suppressing immunosuppressive cell populations and amplifying effector immune responses. The dual modulation of PD-L1 expression and immune cell dynamics provides a strong rationale for the clinical evaluation of Roscovitine in combination with immune checkpoint blockade in NSCLC and potentially other solid tumors.

## Introduction

Over the past decade, immune checkpoint blockade (ICB) targeting the PD-1/PD-L1 axis has transformed cancer therapy, including for non-small cell lung cancer (NSCLC) (1). However, a substantial proportion of patients fail to respond to ICB (2). While PD-L1 expression on tumor cells is often used as a predictive biomarker for response, high PD-L1 levels have paradoxically been associated with poor clinical outcomes (3). PD-L1 binds to PD-1 on tumor-specific T cells, inducing T cell exhaustion and reducing cytotoxic activity against tumor cells (4). This immunosuppressive signaling is amplified by other components of the tumor microenvironment (TME), especially myeloid-derived suppressor cells (MDSCs), which suppress effector T cell responses and are associated with poor responses to ICB across multiple cancer types (5). Importantly, PD-L1 is not only expressed by tumor cells but also by immune-suppressive cells within the TME, including MDSCs (5). Its expression within these populations further contributes to immune evasion and ICB resistance. In addition to its role in immune suppression, PD-L1 signaling has been implicated in promoting tumor cell proliferation, survival, and invasiveness (3), underscoring its dual role in tumor progression and immune escape.

Roscovitine (Seliciclib) is a small-molecule inhibitor that binds competitively to ATP-binding sites and inhibits several cyclin-dependent kinases (CDKs), including CDK1, CDK2, CDK5, CDK7, and CDK9 (6–8). Despite its broad activity, Roscovitine exhibits preferential inhibition of CDK5, with an IC50 of 1.6 µM, and remains the only well-characterized CDK inhibitor with relative selectivity for CDK5 over other CDK family members (9). CDK5, a proline-directed serine/threonine kinase, is aberrantly active in multiple cancer types and has emerged as a key regulator of PD-L1 expression. Unlike classical CDKs, CDK5 is not activated by cyclins but instead by the non-cyclin regulatory proteins p35 and p39 (10). Prior studies have shown that CDK5 enhances PD-L1 expression in medulloblastoma (11), and we have previously demonstrated that genetic knockdown of CDK5 or pharmacological inhibition with Roscovitine increases proteasomal degradation of PD-L1 leading to a reduction in PD-L1 expression in NSCLC (12). In addition to its effects on tumor-intrinsic PD-L1 expression, Roscovitine has been shown to reduce levels of immune effector cells such as eosinophils and neutrophils, leading to reduced inflammation (13). This dual activity suggests that Roscovitine may have therapeutic potential in combination with standard antibody-based ICB in NSCLC, allowing more effective antibody-mediated blockade of PD-L1 by both reducing PD-L1 expression and modulating the inflammatory tumor microenvironment.

In this study, we evaluated combining Roscovitine with anti-PD-1 in a syngeneic, immune-competent mouse model of lung cancer, finding that this combination dramatically suppresses tumor growth and significantly prolongs survival. The effect is partly due to the ability of Roscovitine to enhance anti-tumor immunity by down regulating PD-L1 expression, reducing MDSC levels, and increasing cytotoxic CD8+ T cells and NK cells within the TME. The results suggest that CDK inhibition, and more importantly CDK5 inhibition is a promising strategy to overcome resistance and improve ICB efficacy in NSCLC.

## Materials and methods

### Mice and tumors

All experiments were performed under institutional animal care and use committee (IACUC) protocols, adhering to USDA guidelines. C57BL6/J mice were purchased from The Jackson Laboratory (Bar Harbor, ME) and maintained pathogen-free under UDSA guidelines. Lewis lung cancer (LLC) cells tagged with luciferase (LLC-luc) were from ATCC. These cells are syngeneic in C57/BL6 mice, and were maintained in RPMI medium supplemented with 10% (vol/vol) FBS, 100 units/mL penicillin, and 100 μg/mL streptomycin in a 5% CO_2_ atmosphere at 37°C. LLC-luc cells were inoculated into 10 week-old C57/BL6 mice (5 x 10^4^ cells intrathoracically in 50 μl PBS) to generate orthotopic lung tumors. After 3 days, mice were randomized to experimental groups: i) Roscovitine (Selleckchem, 100 mg/kg i.p., 5 days/wk), ii) mouse anti-PD-1 (BioXcell, Clone RMP1–14; 4 mg/kg i.p., weekly), iii) Roscovitine + mouse anti-PD-1, iv) controls, which received the vehicle used for Roscovitine and an isotype control immunoglobulin (4 mg/kg i.p. weekly). Total body weights were measured on alternate days. Mice were treated for 3 consecutive weeks. Tumors were monitored by bioluminescence imaging using an IVIS Spectrum *in vivo* imager. Differences in tumor growth were analyzed by Student’s t-test.

For high-parameter flow cytometry, LLC-Luc cells (10^5^ per mouse) were orthotopically inoculated into the lungs of C57BL/6 mice. After 18 days, the mice were randomly assigned to one of four experimental groups: i) Roscovitine, ii) mouse anti-PD-1, iii) Roscovitine plus mouse anti-PD-1, and iv) controls, which received the vehicle used for Roscovitine and an isotype control immunoglobulin. After 3 days of treatment, blood was collected, and lung tumors were harvested to examine immune markers using high-parameter flow cytometry.

### High-parameter flow cytometry

Viable cells isolated from the blood and lung tumors of tumor-bearing mice were assessed for frequency of T-cell and myeloid cell subsets (14). PD-L1 levels were measured in circulating immune cells, tumor cells, and tumor-infiltrating myeloid cells. Cell suspensions were stained with antibodies for CD45 (Clone 30-F11), T cell markers – CD4 (Clone GK1.5), CD8 (Clone 53–6.7), or myeloid markers – Ly6G (Clone RB6-8C5), CD11b (Clone M1/70), CD11c (Clone HL3), CCR2 (Clone 475,301), I-A/I-E (clone M5/114.15.2, BioLegend), or PD-L1/CD274 (Clone MIH5, eBioscience). Myeloid derived suppressor cells were gated as M-MDSC CD11b+Ly6GLo and PMN-MDSC CD11b+Ly6GHi, and dendritic cells as CD11b+CD11c+I-A/I-E+. Antibodies were from BD Biosciences unless otherwise specified. The cells were acquired for flow cytometry using the BD FACSymphony A5 cell analyzer, and data was subsequently analyzed with the FlowJo software package (BD Biosciences). **Table 1** below represents specific cell populations identified using flow cytometry based on surface marker expression. Each marker represents a protein (or cluster of differentiation, CD) found on the surface of cells, and the **+/−** symbols indicate whether the marker is expressed or absent.

**Table 1:** Gating strategy for high-parameter flow cytometry.

Cell subsets	Specific gating
NK cells	CD45+CD3-B220-NKp46+NK1-1+
T cells	CD45+CD3+B220-
CD8 T cells	CD45+CD3+B220-CD4-CD8+
M-MDSCs	CD45+/B220-CD3-/CD11b+Ly6G-
PMN-MDSCs	CD45+/B220-CD3-/CD11b+Ly6G+
Dendritic cells (DC)	CD45+/B220-CD3-/CD11b+Ly6G-/CD11c+Ly6C-
M-MDSC Ly6C+Hi	CD45+/B220-CD3-/CD11b+Ly6G-/CD11c-Ly6C+Hi
M-MDSC Ly6C+Lo	CD45+/B220-CD3-/CD11b+Ly6G-/CD11c-Ly6C+Lo
CCR2 in M-MDSCs	CD45+/B220-CD3-/CD11b+Ly6G-/CCR2+
CCR2 in Ly6C+Lo	CD45+/B220-CD3-/CD11b+Ly6G-/CD11c-Ly6C+Lo/CCR2+
PD-L1 in M-MDSCs	CD45+/B220-CD3-/CD11b+Ly6G-/PD-L1+
PD-L1 in DC	CD45+/B220-CD3-/CD11b+Ly6G-/CD11c+Ly6C-/PD-L1+
PD-L1 in Ly6C+Lo	CD45+/B220-CD3-/CD11b+Ly6G-/CD11c-Ly6C+lo/PD-L1+

## Results

### Combining Roscovitine with anti-PD-1 increases anti-tumor efficacy

**Figure 1:**
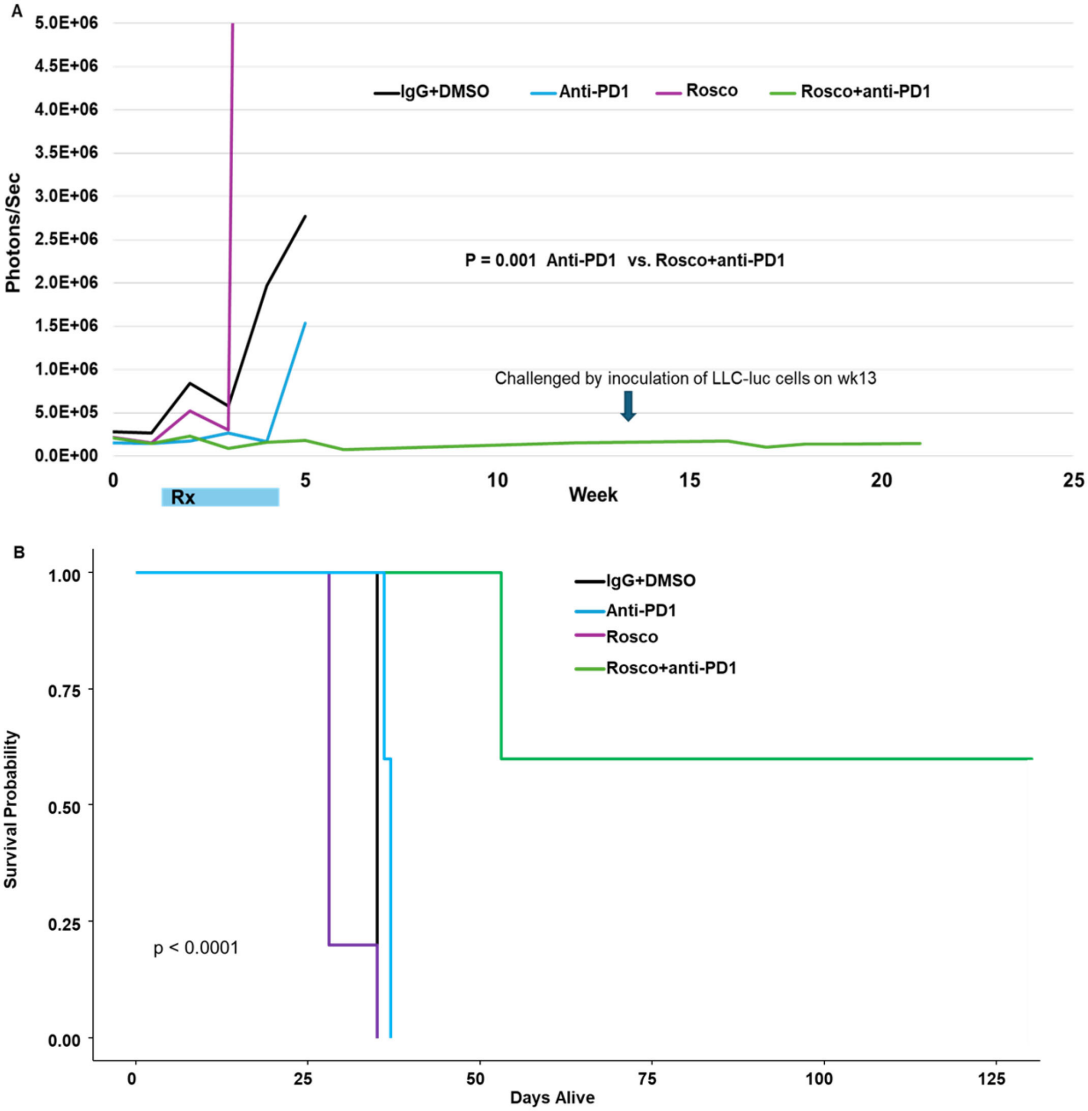
**(A)** Using a syngeneic mouse model of NSCLC, tumor volumes in surviving mice were measured by IVIS luminescence after treatment with Roscovitine (Rosco), anti-PD-1, Rosco + anti-PD-1, or controls. The data are represented as mean photons/sec. **(B)** Percent survival of mice receiving single agents or combinations, and the statistical analysis was done using Brown-Forsythe and Welch ANOVA. The p value represents comparison between anti-PD-1 and Rosco + anti-PD-1. These studies were performed twice independently, and the data presented in the manuscript represent one of these independent experiments.

By reducing PD-L1 levels in cancer cells, Roscovitine is expected to enhance the efficacy of ICB. To assess the therapeutic impact of Roscovitine in combination with ICB, we employed an orthotopic lung cancer model using luciferase-labeled Lewis lung carcinoma (LLC-luc) cells, which are syngeneic in C57/BL6 mice (15, 16). Three days after tumor cell inoculation, mice were randomized into four groups (n=5 per group): i) vehicle control, ii) Roscovitine, iii) anti-PD-1, and iv) Roscovitine plus anti-PD-1. Tumor progression was monitored using bioluminescence imaging. Treatment with either Roscovitine or anti-PD-1 alone did not significantly inhibit tumor growth. In contrast, the combination of Roscovitine and anti-PD-1 led to complete tumor suppression in a substantial proportion of mice (**Figure 1A**). Notably, no tumor regrowth was observed in the combination group even after treatment was stopped at week 4. After 13 weeks, surviving mice were rechallenged with LLC-luc cells without further therapy. None of the rechallenged mice developed tumors (**Figure 1A**), indicating that the combination treatment triggered a robust anti-tumor immune response, resulting in potent and long-lasting anti-tumor immunity. In survival analysis, Roscovitine alone did not improve survival. However, mice receiving combination treatment exhibited significantly extended survival (~100 days longer than vehicle controls) (**Figure 1B**).

### Combination treatment with Roscovitine and anti-PD-1 enhances immune responses by modulating immune cell populations

**Figure 2:**
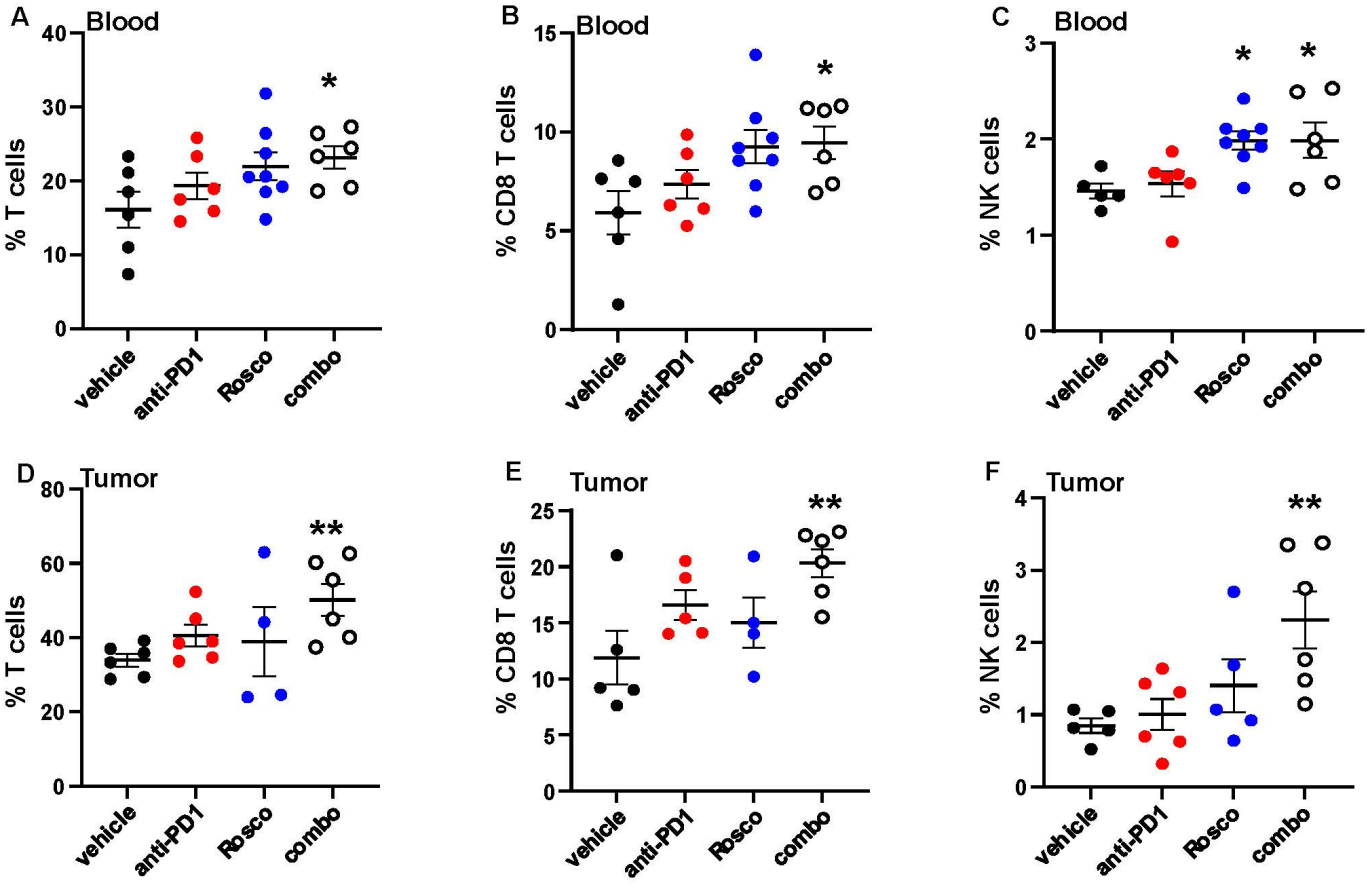
Roscovitine and anti-PD-1 combination modulates immune cell composition in NSCLC tumor-bearing mice. High parameter flow cytometry was used to assess the cellular composition of blood and tumor tissues from mice, using the antibody panels described in **Table 1** in Materials and Methods. **(A)** Percent T cells; **(B)** Percent CD8 T cells; **(C)** Percent NK cells in blood; and **(D)** Percent T cells; **(E)** Percent CD8 T cells; **(F)** Percent NK cells in tumors were determined by flow cytometry. Each dot represents the measurement from an individual mouse, n = 5 or 6 for each group. P values were calculated using Student’s t-test. Statistically significant differences between indicated treatment groups are marked with asterisks (*p < 0.05, **p < 0.005).

Based on these results, we hypothesized that Roscovitine enhances sensitivity to anti-PD-1, at least in part, by increasing the immune response. CDK5, in addition to its role in cancer cells, is also expressed in immune cells and regulates T-cell activation (17). Therefore, following treatment with Roscovitine plus anti-PD-1, we analyzed blood and tumor tissue samples from tumor-bearing mice treated for 3 days with either anti-PD-1, Roscovitine, or the combination for changes in immune cell populations by high-parameter flow cytometry. Immune phenotyping was performed on cells gated for CD45^+^ from both blood and tumor tissues (**Table 1**). The percentage of T cells (gated as CD45^+^CD3^+^B220⁻) increased in both blood and tumor tissues of mice treated with Roscovitine plus anti-PD-1 compared to mice treated with vehicle (**Figures 2A, D**). However, there was no significant increase in T cell percentages in mice treated with either Roscovitine or anti-PD-1 alone. These results indicate that the combination therapy promotes T-cell recruitment or expansion, which is likely to contribute to enhanced anti-tumor immunity. Given the pivotal role of CD8^+^ T cells in cytotoxic immune responses, where they kill tumor cells directly (18), it is also notable that the combination therapy resulted in a higher percentage of circulating and tumor-infiltrating CD8^+^ T cells (gated as CD45^+^CD3^+^B220^-^CD4^-^CD8^+^) (**Figures 2B, E**).

NK cells and CD8^+^ T cells have complementary roles in tumor immunity. NK cells provide rapid, non-specific cytotoxicity against tumor cells, while CD8^+^ T cells deliver a more targeted and sustained response against tumor-specific antigens (19). Both cell types are crucial for controlling tumor growth and enhancing the efficacy of immunotherapies. We observed a significant increase in the percentage of circulating and tumor-infiltrating NK cells (gated as CD45^+^CD3^-^B220^-^NKp46^+^NK1.1^+^) in mice treated with Roscovitine plus anti-PD-1 compared to vehicle (**Figures 2C, F**), suggesting that the combination therapy enhances immune-mediated anti-tumor responses by augmenting both adaptive (T cell) and innate (NK cell) immunity, with pronounced effects in the tumor microenvironment.

### Treatment with Roscovitine, alone or in combination with anti-PD-1, decreases the levels of total MDSCs and specific MDSC subsets

**Figure 3:**
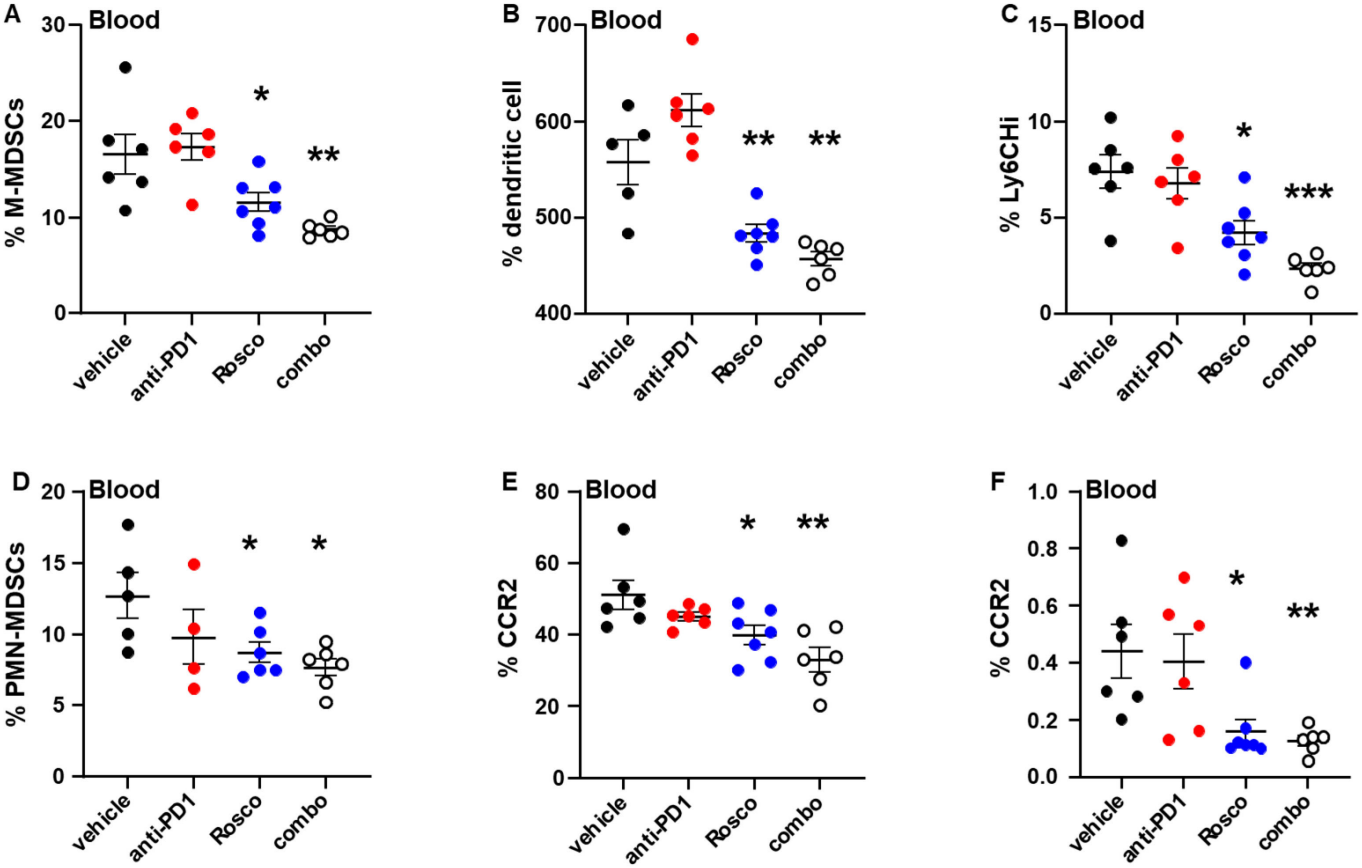
Treatment with Roscovitine, alone or in combination with anti-PD-1, decreases the levels of MDSCs and specific MDSC subsets. **(A–D)** The percentage of circulating monocytic MDSCs [CD11b^+^Ly6C^-^; **(A)** dendritic cells [CD11b^+^Ly6C^-^; **(B)**], M-MDSC subset Ly6CHi [CD11b^+^Ly6G^-^; **(C)**] and the polymorphonuclear MDSC subset PMN-MDSC [CD11b^+^Ly6G^+^; **(D)**] were determined by high parameter flow cytometry. **(E, F)** The percentages of CCR2 in the MDSC subsets CD11b^+^Ly6G^-^**(E)** and in Ly6CLo subset of CD11b^+^Ly6G^-^ cells **(F)** in blood were determined. Each dot represents a measurement from an individual mouse. P values were calculated using Student’s t-test. Statistically significant differences between indicated treatment groups are marked with asterisks (*p < 0.05, **p < 0.005, ***p <.001).

The levels of MDSCs, a heterogeneous group of immature myeloid cells with potent immunosuppressive functions, are elevated in the peripheral blood of cancer patients (20). These cells play a critical role in promoting tumor progression by suppressing T cell activity and facilitating immune evasion (20). Abnormal MDSC accumulation in patients with advanced melanoma is strongly associated with resistance to immunotherapy, as a higher monocyte-like MDSC (M-MDSC) frequency is associated with decreased expansion and activation of tumor-specific T cells (20). Strategies targeting MDSCs have been tested in various tumor types, demonstrating enhanced immunogenicity and, in some cases, reversal of resistance to checkpoint blockade (21). In the present study, treatment with Roscovitine, either alone or in combination with anti-PD-1, led to a significant reduction in circulating MDSCs, including M-MDSCs (CD11b^+^Ly6G^-^) and dendritic cells (CD11c^+^Ly6C^-^), compared to vehicle controls (**Figures 3A, B**). M-MDSCs are characterized by high expression of Ly6C (22). We observed that treatment with Roscovitine, alone or in combination with anti-PD-1, significantly decreased the percentage of the Ly6C^+^ M-MDSC subset Ly6CHi, compared to vehicle (**Figure 3C**). Similarly, polymorphonuclear MDSCs (PMN-MDSCs; CD11b^+^Ly6G^+^) were also significantly down regulated in response to both Roscovitine alone and the combination therapy, compared to vehicle (**Figure 3D**). These findings indicate that Roscovitine, alone or in combination with anti-PD-1, effectively reduces the levels of MDSCs associated with immune suppression (20), potentially enhancing anti-tumor immunity and improving therapeutic outcomes in cancer. MDSCs infiltrate the TME and contribute to the immune-suppressive phenotype typical of many tumors (21). Although the exact mechanism of how MDSCs are recruited to tumor sites is not fully understood, growing evidence suggests that chemokine receptors, especially the chemokine receptor 2 (CCR2), play a key role in this process (23). In the present study, treatment with Roscovitine significantly decreased the percentage of CCR2-expressing myeloid cells, both in M-MDSCs (CD11b^+^Ly6G^-^; **Figure 3E**) and in the M-MDSC subset gated as Ly6CLo (CD11b^+^Ly6G^-^; **Figure 3F**), compared to vehicle. This reduction was further amplified in the combination with anti-PD-1 (**Figures 3E, F**), suggesting that these treatments selectively deplete or inhibit the recruitment and function of M-MDSCs. By simultaneously reducing CCR2 expression and MDSC populations, Roscovitine disrupts critical pathways involved in MDSC-mediated immune suppression, thereby promoting enhanced anti-tumor immunity when used in combination with ICB.

### Roscovitine treatment reduces PD-L1 expression in circulating and tumor-infiltrating MDSC subsets

**Figure 4:**
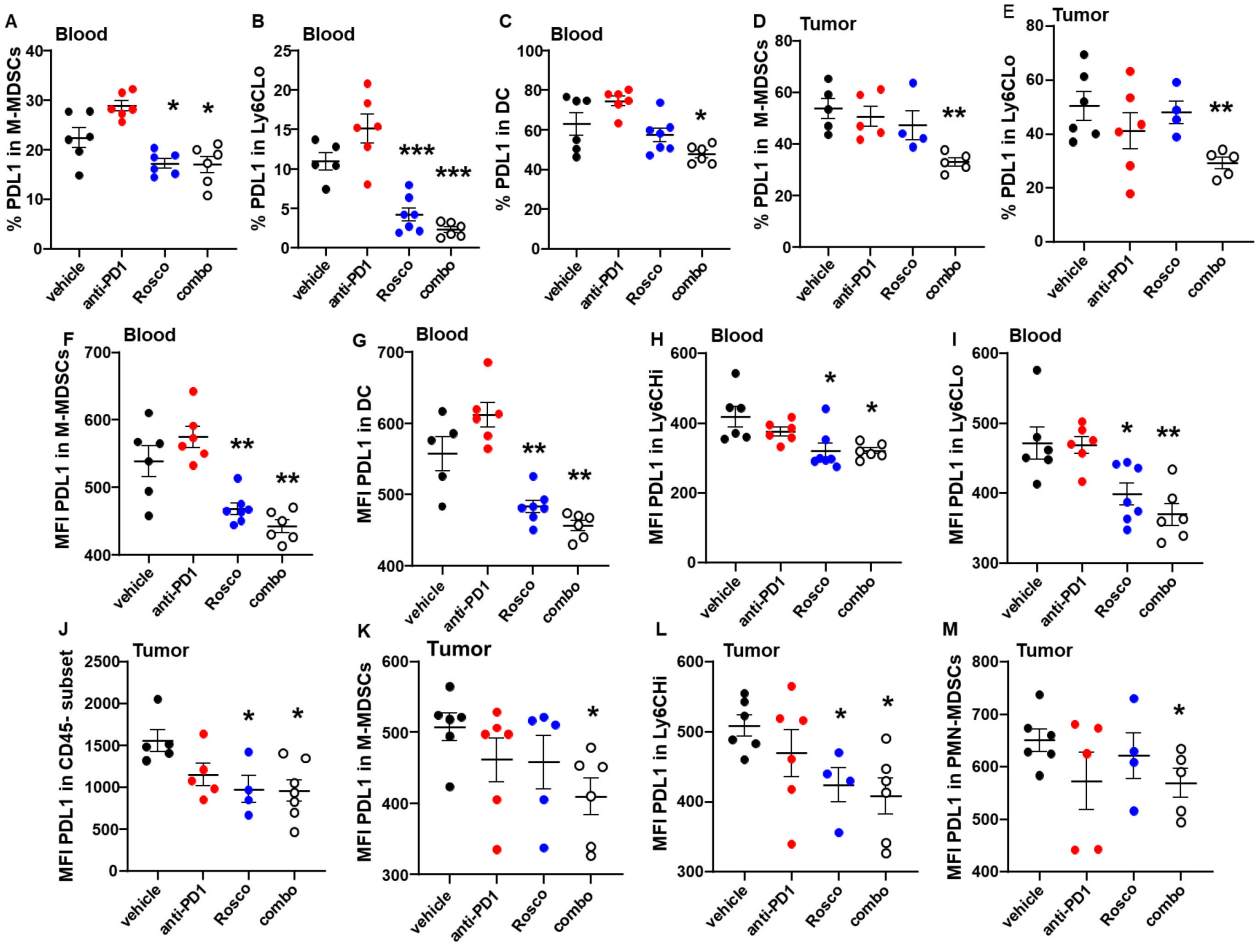
Roscovitine treatment reduces PD-L1 expression in circulating and tumor-infiltrating MDSC subsets. **(A–C)** The percentages of PD-L1 expressed in MDSC subsets in blood from tumor-bearing mice determined by high parameter flow cytometry. **(D, E)** The percentages of PD-L1 expressed in tumor-infiltrating MDSCs. **(F–I)** The mean fluorescence intensity (MFI) of PD-L1 in MDSC subsets from blood. **(J)** The MFI of PD-L1 in CD45 negative populations in the tumors. **(K–M)** The MFI of PD-L1 in tumor-infiltrating MDSC subsets. Each dot represents a measurement from an individual mouse. P values were calculated using Student’s t-test. Statistically significant differences between indicated treatment groups are marked with asterisks (*p < 0.05, **p < 0.005, ***p <.001).

PD-L1 is highly expressed in MDSCs, leading to immunosuppression and development of resistance to ICB (20). Based on our previous finding that Roscovitine decreases PD-L1 in tumor cells (12), we hypothesized that it would enhance the sensitivity to anti-PD-1, at least in part by increasing the immune response by specifically decreasing PD-L1 levels. We observed that treatment with Roscovitine significantly decreased PD-L1 expression in both circulating (**Figures 4A–C**) and tumor-infiltrating (**Figures 4D, E**) MDSC subsets compared to vehicle controls. Analyzing the PD-L1-associated mean fluorescence intensity (MFI) in addition to the percentage of PD-L1-expressing cells provides a more comprehensive understanding of how PD-L1 expression is regulated at both qualitative and quantitative levels. Therefore, we also evaluated the MFI of PD-L1 in various MDSC subsets, including M-MDSCs, Ly6CHi, and Ly6CLo cells. After treatment, the MFI of PD-L1 significantly decreased in all these populations in circulating MDSCs (**Figures 4F–I**). Consistent with our previous findings that Roscovitine decreases PD-L1 levels in tumor cells (12), treatment with Roscovitine significantly decreased the PD-L1-associated MFI in tumor-derived CD45- populations, which consist primarily of tumor cells devoid of immune cells (**Figure 4J**). Importantly, the MFI of PD-L1 was also decreased in tumor MDSC subsets, including M-MDSCs, LyC6Hi, and PMN-MDSCs after treatment with Roscovitine compared to vehicle controls (**Figures 4K–M**). Therefore, Roscovitine may reduce the immunosuppressive capacity of MDSC subsets by down regulating PD-L1 expression, thereby enhancing the therapeutic efficacy of anti-PD-1.

## Discussion

The combination of Roscovitine with anti-PD-1 demonstrates strong anti-tumor efficacy and survival benefit, as shown in our immune-competent mouse model of lung cancer. These findings align with prior studies indicating that Roscovitine can reduce PD-L1 levels in cancer cells, enhancing the effectiveness of ICB (12). PD-L1 contributes to immune evasion by tumor cells by inhibiting effector T-cell function (1). Our study shows that adding Roscovitine to anti-PD-1 therapy leads to complete suppression of tumor growth, even after treatment cessation. Remarkably, re-challenged mice previously treated with the combination showed no tumor recurrence, demonstrating that combination treatment dramatically increases anti-tumor immune responses, in part because of the impact of the combination therapy on immune cell populations.

To model lung cancer in an immunocompetent setting, we employed LLC-luc cells implanted orthotopically in C57BL/6 mice. The LLC model is among the most widely used and well-characterized syngeneic models for NSCLC. LLC-luc cells, derived from spontaneous lung tumors, allow for immune interaction analysis in a native lung microenvironment (16). Importantly, this model is known to exhibit resistance to ICB monotherapy but can respond to combination strategies, such as ICB plus radiotherapy (16, 24), underscoring its relevance for testing approaches aimed at overcoming ICB resistance. A recent study showed that the molecular profile of LLC closely resembles that of human lung adenocarcinoma, supporting its relevance as a lung cancer model (25).

CDK5 plays a central role in regulating immune checkpoints in tumor cells. Studies in medulloblastoma showed that loss of CDK5 leads to sustained expression of PD-L1 transcriptional repressors, resulting in reduced PD-L1 levels on tumor cells (11). Similarly, in our previous work, genetic down regulation of CDK5 or treatment with Roscovitine in NSCLC decreased PD-L1 expression by promoting its degradation via the E3 ligase FBXO22 (12). These findings underscore the pivotal role of CDK5 in maintaining immune suppression within the tumor microenvironment, thereby facilitating resistance to ICB. Inhibition of CDK5 with Roscovitine disrupts these immune checkpoint pathways, reducing PD-L1 levels and alleviating tumor-driven immune suppression. In the present study, we observed that Roscovitine plus anti-PD-1 increased T-cell recruitment and expansion, especially CD8^+^ T cells, in both blood and tumor tissues. CD8^+^ T cells are essential for cytotoxic responses against tumor cells (16). Moreover, the increase in tumor-infiltrating NK cells, which complement CD8^+^ T cells by providing rapid, non-specific cytotoxicity, further supports the synergistic effects of this therapy. Together, these observations suggest that Roscovitine plus anti-PD-1 enhances both adaptive and innate immune responses, thus improving anti-tumor immunity and potentially overcoming the limitations of anti-PD-1 monotherapy.

MDSCs contribute to an immunosuppressive tumor microenvironment by inhibiting T-cell function and resistance to ICB, thus facilitating immune evasion (20, 21). In the present study, Roscovitine, alone or in combination with anti-PD-1, significantly decreased circulating MDSCs, including M-MDSCs and PMN-MDSCs, subtypes known to be involved in immune suppression (21). Furthermore, the combined therapy reduced the expression of chemokine receptor CCR2, which is implicated in MDSC recruitment to tumors (23). These reductions in MDSC populations and CCR2 expression are likely to diminish immune suppression within the tumor microenvironment, enhancing the therapeutic impact of anti-PD-1. PD-L1 is upregulated in tumor cells and various immunosuppressive cells within the TME, including MDSCs (20–22). Importantly, we observed that treatment with Roscovitine led to a significant reduction in PD-L1 expression in circulating and tumor-infiltrating MDSC populations, as well as in CD45^-^ tumor cells, which represent non-immune cells within the tumor. Reduced PD-L1 expression in these cells decreases the immune-inhibitory signal that limits T cell function, alleviating MDSC-mediated immunosuppression and promoting a sustained anti-tumor immune response.

Our results show that although Roscovitine alone induces a favorable TME by lowering PD-L1 expression, increasing CD8^+^ and NK cell infiltration, and reducing CCR2^+^ MDSCs, these changes did not decrease tumor growth or increase survival. This is likely because the recruited immune cells remain functionally suppressed through PD-1 receptor engagement; that is, PD-1 on T cells and NK cells continue to bind PD-L1 on tumor and myeloid cells, delivering an inhibitory signal that limits their cytotoxic activity (26). Anti-PD-1 therapy disrupts this suppressive interaction, restoring effector function and allowing the Roscovitine-primed immune cells to enhance anti-tumor responses. Although Roscovitine is a first-generation CDK inhibitor, its immunomodulatory effects in our study were achieved at doses lower than those required for its anti-proliferative activity. Specifically, we observed that administering three, non-toxic doses of Roscovitine was sufficient to downregulate PD-L1 and reduce CCR2^+^ MDSCs, indicating that these immune-modulating effects occur within a dosing range that avoids toxicity. This suggests that the immune-stimulating properties of Roscovitine may be therapeutically leveraged within a safer window, particularly when used in combination with checkpoint blockade rather than as a cytotoxic monotherapy. Thus, the mechanistic insights gained from our study may guide the future integration of more selective CDK inhibitors with ICB.

In summary, combining Roscovitine with anti-PD-1 offers a promising strategy to enhance ICB by modulating immune cell populations, reducing MDSC-driven suppression, and decreasing PD-L1 expression in the tumor microenvironment. These combined effects contribute to improved anti-tumor responses and increased survival, highlighting the potential of integrating Roscovitine with anti-PD-1 therapy. While we observed decreased CCR2^+^ MDSCs in circulation, additional studies are needed to fully characterize how Roscovitine affects MDSC recruitment and suppressive function. Future studies might explore more comprehensive immune profiling to elucidate the pathways by which Roscovitine modulates immune cell populations, including potential links between CDK5 inhibition and immune regulation. Such studies will be critical for validating the broader application of Roscovitine in overcoming ICB resistance across diverse cancer types in which immunosuppressive myeloid populations and PD-L1 expression limit therapeutic response.

## Data Availability

The original contributions presented in the study are included in the article/**Supplementary Material**. Further inquiries can be directed to the corresponding author.
