# Supplementary figures and images for: The CDK inhibitor Roscovitine enhances the therapeutic efficacy of anti-PD-1 in non-small cell lung cancer
Source: Front Oncol. 2026 Jan 5;15:1745967. doi: 10.3389/fonc.2025.1745967 (PMC12812617; doi:10.3389/fonc.2025.1745967)

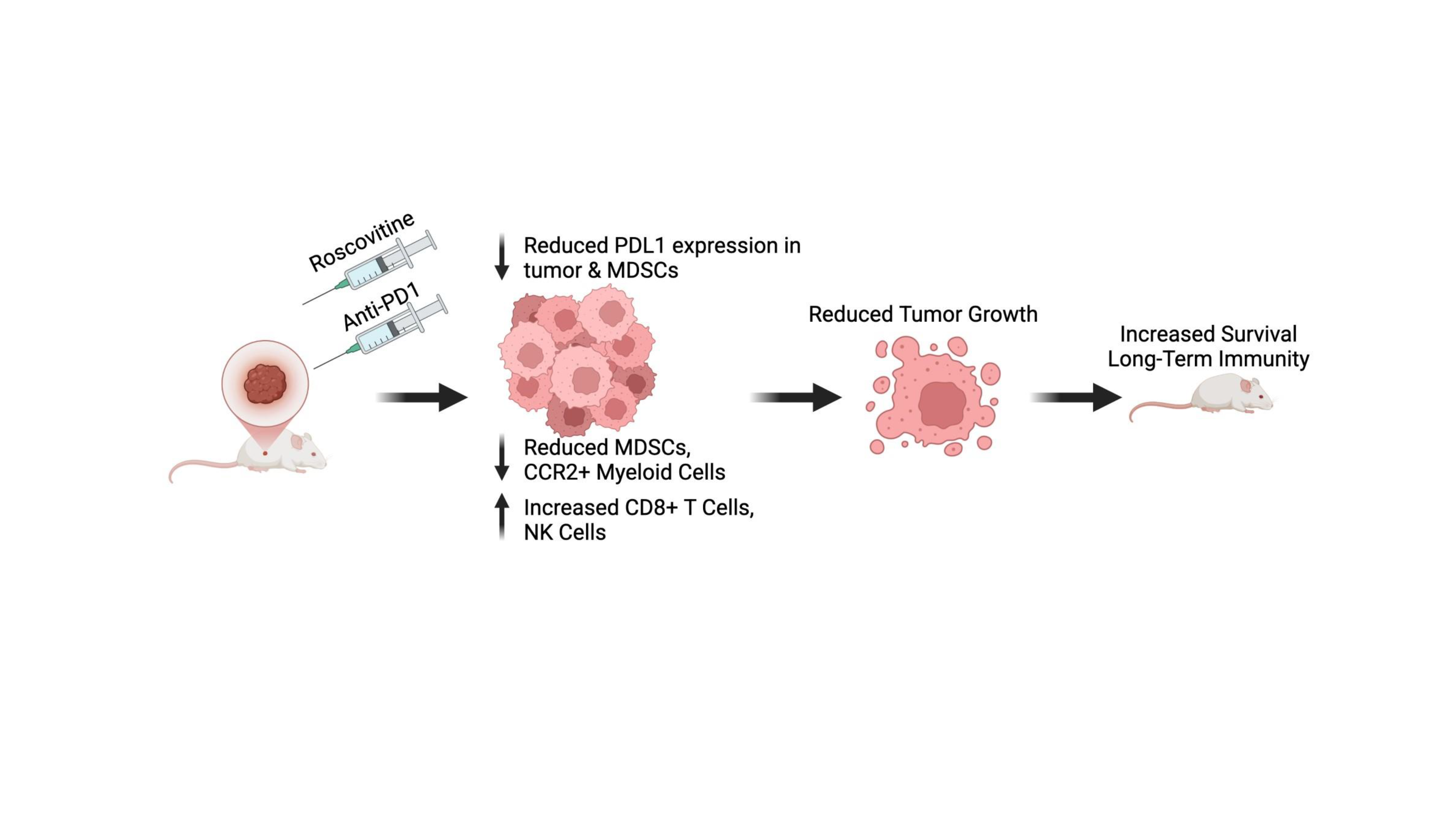

Supplement: Supplementary file 1 [file Image1.tif]
